# Controlling the HIV/AIDS epidemic: current status and global challenges

**DOI:** 10.3389/fimmu.2012.00250

**Published:** 2012-08-14

**Authors:** Thorsten Demberg, Marjorie Robert-Guroff

**Affiliations:** Vaccine Branch, Section on Immune Biology of Retroviral Infection, National Cancer Institute, National Institutes of HealthBethesda, MD, USA

**Keywords:** animal model, ART treatment, global strategies, HIV pandemic, prevention, vaccine development

## Abstract

This review provides an overview of the current status of the global HIV pandemic and strategies to bring it under control. It updates numerous preventive approaches including behavioral interventions, male circumcision (MC), pre- and post-exposure prophylaxis (PREP and PEP), vaccines, and microbicides. The manuscript summarizes current anti-retroviral treatment options, their impact in the western world, and difficulties faced by emerging and resource-limited nations in providing and maintaining appropriate treatment regimens. Current clinical and pre-clinical approaches toward a cure for HIV are described, including new drug compounds that target viral reservoirs and gene therapy approaches aimed at altering susceptibility to HIV infection. Recent progress in vaccine development is summarized, including novel approaches and new discoveries.

## Introduction

Beginning with the FDA approval of AZT in 1987 for HIV treatment and the subsequent development of combination therapies, millions of HIV infected people have been saved from progression to AIDS, and their quality of life has improved dramatically (Broder, 2010). An outgrowth of the successful advances in AIDS treatment regimens is the recent finding that early initiation of antiretroviral therapy (ART) can reduce HIV transmission by 96% (Cohen et al., 2011). Moreover, mother-to-child transmission can be prevented using anti-HIV drugs, as recently reviewed for low- and middle-income countries (Siegfried et al., 2011; Tudor Car et al., 2011; Santos et al., 2012). The challenges of implementing such “treatment as prevention” strategies are many, however, as reviewed here. Overall, in addition to pursuing new developments in treatment, all available preventive measures as well as continued research efforts toward an efficacious HIV vaccine should be employed to effectively control the HIV/AIDS pandemic.

## Facts and finances: the global challenge of HIV infection

Human immunodeficiency virus (HIV) infection and the incurable disease, acquired immunodeficiency syndrome (AIDS), present major global health problems. Approximately 34 million people worldwide are currently living with HIV, 1–1.2 million of these in the United States (Table 1). The overall US HIV infection rate has remained steady since the 1990s with around 49,000 cases per year, the majority in men having sex with men (MSM) (Moore, 2011; Morris and Little, 2011; Prejean et al., 2011). Moreover, the global HIV prevalence is expected to increase within the next 10 years (Yehia and Frank, 2011), indicating that action on a national and international level is needed. The current standard of care is ART which requires daily medication to prevent disease progression and re-emergence of the virus. However, co-infection of HIV infected patients with tuberculosis or hepatitis as well as sexually transmitted diseases, together with problems in health care systems and infrastructure, have an impact on treatment success (Beauliere et al., 2010; Obiako and Muktar, 2010; Birbeck et al., 2011; Boesecke and Vogel, 2011; Finlayson et al., 2011).

**Table 1:** **HIV/AIDS: facts and finance**.

**Prevalence**	**Currently HIV infected**	**Newly infected (2010)**	**AIDS deaths**	**References**
US	1.2 million	49 thousand	17 thousand (2009)	Prejean et al., 2011, 1
Global	34 million	2.7 million	1.8 million (2010)	2
**Treatment costs**	**Per person/year**	**Currently infected/year**	**Newly infected/year**	
US	$10–$60 thousand	$14–$23 billion^a^	$1.47 billion^b^	Fleishman et al., 2010; Gebo et al., 2010; Chesson et al., 2011; Hill et al., 2011; Krentz and Gill, 2012
Treatment through the global AIDS fund	First line ART: $487, second line ART: $1521	3.5 million people treated (2011): ~$1.9 billion	–	Stover et al., 2011

a Estimated cost for treatment of STDs and HIV.

b Estimated using $30,000 per person, per year.

1. http://www.cdc.gov/hiv/resources.factsheets/us.htm

2. http://www.who.int/hiv/data/2011_epi_core_en.png

ART is expensive as illustrated by costs in the US that average $30,000/year/patient and are similarly high in European countries (Table 1) (Fleishman et al., 2010; Gebo et al., 2010; Colombo et al., 2011; Hill et al., 2011; Krentz and Gill, 2012). Despite multi-tier pricing models (Wirtz et al., 2009), global costs for treatment are high (Table 1) and current predictions call for over 1.7 billion dollars per year to fight HIV in low- and middle-income countries (Nunn et al., 2007; Stover et al., 2011). Moreover, the future of outside funding for treatment (e.g., PEPFAR, the President's Emergency Plan for AIDS Relief) under current global economic conditions is tenuous, as a drop in funding due to the weakened global economy has been reported with consequences for programs and patients in Africa (Voelker, 2011; Wasswa, 2011). Worldwide, treatment costs impact families, economies and health care systems. In the US alone, therapy for STDs and HIV cost from 14 to 23 billion dollars a year (Chesson et al., 2011). HIV/AIDS is associated with poverty, lower education and minority populations (Torrone et al., 2010; Vermund et al., 2010; Dang et al., 2011; Espinoza et al., 2012). Only 15–17% people living with HIV have private insurance and nearly 30% in the US do not have any coverage (Espinoza et al., 2012; http://healthcare.gov/news/factsheets/affordable_care_act_people_hiv_aids.html). Since up to 21% of these groups would likely be starting treatment in an advanced disease state due to unawareness, late detection of infection (Moore, 2011; Yehia and Frank, 2011; Espinoza et al., 2012) or being undocumented (Dang et al., 2011) costs for therapy would be higher. Measures to prevent HIV infection at the outset would provide a significant financial benefit by lowering health care costs. Overall, finding measures to prevent HIV infection or affect a cure are of highest importance for the broadest benefit to public health (Long and Owens, 2011).

## Worldwide implementation of art

Effective treatment enables HIV infected individuals to be productive members of society, a critical economic benefit (Resch et al., 2011). However, making treatment worldwide readily available is a challenge. Aside from high costs (Table 1), successful initiation and maintenance of AIDS therapy in emerging countries and the developing world can be limited by political and sociological factors including instability of governments and health care systems, transportation problems and poor nutrition (Beauliere et al., 2010; Obiako and Muktar, 2010; Birbeck et al., 2011). If disease is diagnosed late as often occurs in developing countries, treatment success can be diminished due to late stage AIDS, HIV-associated dementia and opportunistic infection requiring additional, often unavailable, treatment. Social barriers such as personal and family shame or blame and societal stigma, often paired with insufficient education on HIV/AIDS, can also prevent individuals from seeking treatment (Amuri et al., 2011; Dang et al., 2011; Winskell et al., 2011; Espinoza et al., 2012; Steward et al., 2012).

The overall benefits outweigh by far the side effects of ART medications. However, in the course of treatment new problems surface and medical care has to be adjusted accordingly (Llibre et al., 2009) which can be difficult in the developing world. ART can induce immune reconstitution inflammatory syndrome upon initiation, making the monitoring of CD4 T-cells, viral load, liver, and kidney function crucial for successful treatment (Kalyesubula and Perazella, 2011; Keiser et al., 2011; Barber et al., 2012). Further, there is a tendency for HIV infected patients on ART to show increased kidney damage in addition to HIV associated nephropathy and cardiovascular disease. An increased risk for myocardial infarct has been attributed to the protease inhibitors lopinavir/ritonavir and the transcriptase inhibitor abacavir (Fichtenbaum, 2010; Islam et al., 2012a,b; Maggi et al., 2012). Markers of systemic inflammation (c-reactive protein, IL-6), coagulation, and renal function are elevated in HIV infected patients independent of successful suppression of viremia (Neuhaus et al., 2011; Bastard et al., 2012). The elevated inflammatory markers can indicate ongoing low level viral replication in tissues only minimally reached by ART and subsequent cell-to-cell viral spread, as ART does not completely reverse T-cell activation (D'Ettorre et al., 2011). In addition to T-cell activation, B-cell abnormalities and loss of memory B-cells persist despite long-term treatment with ART (Chong et al., 2004; Moir and Fauci, 2009). B-cell lymphomas and other cancers (e.g., squamous cell carcinoma) are increasing in HIV infected patients (Carbone et al., 2009; Epeldegui et al., 2010; Gervaz et al., 2011; Tyerman and Aboulafia, 2012) requiring additional treatment.

First line combination therapy, consisting of one non-nucleoside reverse transcriptase inhibitor (NNRTI) and two nucleoside reverse transcriptase inhibitors (NRTI) as recommended by the WHO, is often the only available form of treatment in low-income countries (www.who.int/hiv/pub/arv/adult2010/en/index.html). As of December, 2010, in 45 low- and middle-income countries excluding the Americas, of the 81% of patients receiving treatment, 97% were on first-line regimens (>54% on Nevirapine, Lamivudine and Stavudine, or Zidovudine) and only 2.9% on significantly more costly second line treatment regimens (www.who.int/hiv/pub/progress_report2011/en/). As a result, this can lead to the emergence of viral resistance to first line treatment. To combat this problem, frequent monitoring of viral loads and strict treatment adherence to the drug regimen are important, but can be problematic (Birbeck et al., 2011). Over time despite treatment adherence and maintenance of viral load suppression, resistance develops in about 30% of patients. Not only does this result in treatment failure, the resistant viruses can be transmitted, cause new infections that are not controlled by the existing drug regimen (Hamilton et al., 2012; von Wyl et al., 2011), and jeopardize future treatment options (Chamberland et al., 2011). Especially, concerning is the frequency of viral resistance to first-line ART that arises among children in resource poor countries (Sigaloff et al., 2011). Resistance mutations vary depending on the type of drug regimen. However, regardless of the mechanism, resistance to ART develops inexorably, showing the urgent need not only for new treatments but also alternative preventive measures.

## Additional approaches to prevent infection

In spite of the dramatic reports of preventing HIV transmission using early ART, other studies have suggested that testing and early treatment alone might not significantly reduce HIV transmission in the near future, as predicted by a study in Washington, DC (Walensky et al., 2010), a current “hot-spot” of HIV infection (Greenberg et al., 2009; Carr et al., 2010; Hanna et al., 2011). To be prudent, additional measures, of which there are several, would enhance the likelihood of successful prevention. Among the easiest is syringe and needle exchange programs. HIV is most commonly transmitted by sexual contact. However, transmission also occurs among intravenous drug users (IDU) via needle and syringe sharing (Nacopoulos et al., 2010; Vlahov et al., 2010). In the US approximately 12% of all new HIV infections per year occur in IDU (Adimora and Auerbach, 2010). Syringe and needle exchange programs provide a simple, inexpensive preventive measure in this population (Adimora and Auerbach, 2010; Nacopoulos et al., 2010; Vlahov et al., 2010).

Sexual transmission of HIV is best prevented by practicing safe sex or abstinence, both confounded by multifaceted behaviors. Failure to practice safe sex may be due to lack of knowledge about HIV infection and treatment, and perhaps the assumption that ART cures HIV (Owoaje et al., 2011; Smith et al., 2011). Other factors include gender inequity of woman, particularly in negotiating condom use, and partner violence (Jewkes et al., 2010; East et al., 2011; El-Bassel et al., 2011; Fair and Vanyur, 2011; Gakumo et al., 2011; Swan and O'Connell, 2011). Additionally, alcohol and substance abuse influence personality traits and risk behavior, and lower the frequency and ability to negotiate condom use (Tapert et al., 2001; Kogan et al., 2010; Finlayson et al., 2011; Hagger-Johnson et al., 2011; Maisto et al., 2012; Rehm et al., 2012). Selected clinical trials that address behavioral modification are listed in Table 2. Behavioral modification is difficult, however, and mixed results have been obtained. Of five investigated interventions (billboards, peer education, magazines, radio broadcasts, and public outreach events) the latter three were significantly correlated with increased condom use, but the overall influence on behavior was limited (Hsu et al., 2012). In another study, behavioral interventions among 725 HIV-positive women had no effect on increased condom use (Carvalho et al., 2011). Behavioral interventions in the workplace, including voluntary counseling and testing to reduce risky sexual behavior, found no reduction in HIV incidence rate or frequency of unprotected sex, although, education did reduce unprotected sex, STDs and sex with commercial sex workers (Ojo et al., 2011).

**Table 2:** **Selected clinical trials targeting behavior modification as a measure to prevent HIV infection**.

**Behavioral intervention/counseling**	**Status**	**Phase**	**Target group**	**Alternate study ID**	**Approach**	**References**
NCT00000931	Complete	III	MSM	HIVNET 015 EXPLORE	Behavioral intervention	Koblin et al., 2004; (IIB trial results)
NCT00710060	Complete	III	At-risk populations	DAHBR 9A-ASGT	Community-level prevention program	Rotheram-Borus et al., 2011
NCT00729391	Active	III	Women	R01HD058320	Voluntary counseling and testing; equal attention control group	–
NCT00859144	Not yet recruiting	III	Urban youth	CHAMPions, DAHBR 9A-ASPA	Behavioral intervention: be proud! be responsible!, becoming a responsible teen (BART)	–
NCT00279799	Unknown	III	African American females (age 14–20)	816-2003	AFIYA HIV prevention intervention	–
NCT00310973	Complete	II/III	MSM	DAHBR 9A-ASPG	Educational counseling	Kelly et al., 2006
NCT01152281	Recruiting	II/III	At risk populations	Selective exposure	Meta-intervention to increase retention in prevention counseling	–

More information about the individual trials can be found using the NCT number and the following databases: www.aidsinfo.nih.gov/clinical-trials; www.clinicaltrials.gov; http://www.avac.org.

To augment safe sex practices and abstinence, oral/systemic pre-exposure prophylaxis (PREP) has been shown effective in preventing HIV infection both in non-human primates and humans (Tsai et al., 1994a,b; Grant et al., 2010; Myers and Mayer, 2011). It must be used consciously and before sexual activity in order to achieve a sufficient inhibitor concentration. Reduction in HIV acquisition is dependent on adherence to PREP use and ranges from 21 to 73% (Krakower and Mayer, 2011; Naswa and Marfatia, 2011). Factors associated with PREP failure are similar to those of condom use, including failure to have the medication on hand and adherence (Golub et al., 2010; van der Straten et al., 2012). Questions concerning the implementation of PREP and guidelines for its use are both scientific (e.g., oral vs. mucosal) and policy related (e.g., insurance coverage of treatment expenses) (Myers and Mayer, 2011; Naswa and Marfatia, 2011). Current cost estimates for PREP for 100,000 high risk persons in the US are over $1 billion a year ($900 in medication costs per month) (Leibowitz et al., 2011), making the implementation of PREP in tough economic times unlikely despite potential long-term benefits (Koppenhaver et al., 2011). Further, emergence of drug resistance is a potential pitfall for PREP users but might have only a negligible impact as shown for MSM in the UK (Dolling et al., 2011).

Post-exposure prophylaxis (PEP) or post-exposure prophylaxis following sexual exposure (PEPSE) must be used conscientiously and requires a long follow up interval of up to 6 months post-exposure as recommended by the CDC (www.cdc.gov/mmwr/preview/mmwrhtml/rr5402a1.htm) (McCarty et al., 2011). PEPSE awareness in high risk groups has increased significantly, but its use in general is low (Zablotska et al., 2011). However, PEP after accidental exposure (e.g., needle stick injury) or for victims of sexual assault and rape has a proven track record of preventing infection. As for PREP, timing is critical. Treatment should not be delayed more than 72 h post exposure without a significant chance of infection. Table 3 lists selected clinical trials for PREP and PEP. For further information on PREP and PEP methods, strategies, costs, etc. see Fernandez-Montero et al. (2012).

**Table 3:** **Selected clinical trials investigating PrEP and PEP for prevention of HIV infection**.

**Trial identifier**	**Status**	**Phase**	**Target group**	**Alternate study ID**	**Component**	**Comment/references**
**PrEP**						
NCT00458393	Active	III	MSM	Peru PrEP, iPREX	Emtricitabine/tenofovir disoproxil fumarate	44% efficacy; Grant et al., 2010
NCT00931346	Complete	I/II	HIV discordant couples	IAVI E002	Emtricitabine/tenofovir disoproxil fumarate	–
NCT00557245	Active	III	HIV discordant couples	32528-A, IND 75,365	Emtricitabine/tenofovir disoproxil fumarate	–
NCT01473472	Not yet recruiting	III	MSM	IPERGAY	Emtricitabine and tenofovir disoproxil fumarate (truvada)	–
NCT01275443	Recruiting	I	People at risk for HIV	SSAT 040	TMC278 (rilpivirine), long-acting formulation	–
**PEP**						
NCT00594646	Complete	IV	High-risk sexual contact	MK PEP 2007	Fixed-dose tenofovir and raltegravir	–
NCT00385645	Complete	IV	Accidental HIV exposure	DATEM-PEP	AZT-3TC + lopinavir-ritonavir vs. ZT-3TC + atazanavir	–
NCT01140880	Recruiting	II	MSM	MC08-LA-710-FRI	Emtricitabine and tenofovir disoproxil fumarate (truvada)	–
NCT00949234	Recruiting	II	High-risk sexual contact	PQUAD	Tenofovir + emtricitabine, lopinavir/ritonavir	–
NCT01087840	Recruiting	IV	MSM	RAL-NPEP	Raltegravir	–

More information about the individual trials can be found using the NCT number and the following databases: www.aidsinfo.nih.gov/clinical-trials; www.clinicaltrials.gov; http://www.avac.org.

Microbicide development for HIV prophylaxis is another active area of research. A number of microbicide candidates have been tested in clinical trials (Table 4), but few have shown efficacy, and some have possibly enhanced HIV acquisition (Tao et al., 2008; Abdool Karim et al., 2010, 2011; Pirrone et al., 2011, 2012). The gel base for microbicides must be chosen cautiously, as some can cause epithelial damage facilitating HIV infection (Begay et al., 2011). The latest microbicides containing Tenofovir, a reverse transcriptase inhibitor, have shown promising but modest efficacy, as in the CAPRISA trial (Abdool Karim et al., 2010). However, a recent clinical trial (VOICE, MTN-003) evaluating daily use of a vaginal gel containing Tenofovir in over 5000 woman was discontinued, as interim analysis showed no difference in HIV incidence rate compared to use of a placebo gel (Table 4). Among new approaches, evaluation of topical 2-Hydroxypropyl-beta-cyclodextrin has shown promise in an animal model (Ambrose et al., 2008), as have live lactobacilli engineered to secrete cyanovirin (Lagenaur et al., 2011). The latter approach if successful might alleviate problems of adherence by reducing the need for application just prior sexual exposure. In the Carraguard trial self-reported microbicide use was 96%, but applicator testing revealed actual adherence was only 42% (Skoler-Karpoff et al., 2008). Microbicides under development are incorporating other ART components such as integrase, protease or reverse transcriptase inhibitors. Antibodies are also being considered (Selhorst et al., 2011). A major drawback of current microbicide clinical trials is that efficacy is only tested in heterosexual woman, not in high risk MSM where the majority of new infections occur in the US (Abdool Karim et al., 2010; Krakower and Mayer, 2011; Prejean et al., 2011).

**Table 4:** **Selected clinical trials of microbicides**.

**Microbicides**	**Status**	**Phase**	**Type of prevention**	**Alternate study ID**	**Component**	**Comment/references**
NCT00262106	Complete	III	Gel, vaginal	MDP301, ISRCTN64716212	PRO 2000/5 gel 0.5% and 2%	Not efficacious; McCormack et al., 2010
NCT00153777	Terminated	III	Gel, vaginal	CONRAD, C03-090	Cellulose sulfate gel (6%)	No protection, possible enhancement; Van Damme et al., 2008
NCT00213083	Unknown	III	Gel, vaginal	Population council #322	Carraguard (PC-515)	No protection; Skoler-Karpoff et al., 2008
NCT00441298	Complete	II	Gel, vaginal	CAPRISA 004	Tenofovir 1% gel	39% efficacy; Abdool Karim et al., 2010
NCT00705679	Active	II	Gel, vaginal and ART oral	VOICE, MTN-003	Tenofovir 1% vaginal gel, emtricitabine/tenofovir disoproxil fumarate	Vaginal gel arm halted, no efficacy; 1
NCT00740584	Complete	I/II	Gel, vaginal	SPL7013-003, DAIDS ES number 10730	3% SPL7013 gel (VivaGel)	Price et al., 2011
NCT00850837	Recruiting	I	Gel, vaginal	AF 020	Acidform lubricant (Amphora)	–
NCT01283360	Recruiting	I	Gel, rectal	CONRAD, 1009001	Tenofovir 1% gel	–

More information about the individual trials can be found using the NCT number and the following databases: www.aidsinfo.nih.gov/clinical-trials; www.clinicaltrials.gov; http://www.avac.org.

1. http://www.niaid.nih.gov/news/newsreleases/2011/Pages/VOICEdiscontinued.aspx

Male circumcision (MC) has been recognized fairly recently as a potential preventive measure, but its overall impact may depend on the target population. Multiple trials in Africa have shown efficacy of MC in reducing HIV acquisition in heterosexual men by 38–66%, with uncircumcised men showing an 4-fold higher infection risk in sub-Saharan Africa (Siegfried et al., 2009; Gebremedhin, 2010; Wamai et al., 2011) (Table 5). However, the impact of MC for MSM is unknown (Smith et al., 2010), as is the overall population of MSM in sub-Saharan Africa. It has been estimated that HIV transmission among MSM accounts for 0.6–16% of infections, depending on the country (Wamai et al., 2011). Newer data suggest that MC among MSM in the US, where the overall prevalence of MC already ranges from 43 to 80%, would be of limited benefit (Gust et al., 2010; Jozkowski et al., 2010; Smith et al., 2010; Wei et al., 2011). A protective effect was seen in a subgroup of MSM reporting an insertive role, but overall, in 21 observational studies with 71,693 MSM participants, MC did not prevent HIV transmission (Wiysonge et al., 2011), probably because it has no effect on transmission through receptive anal intercourse.

**Table 5:** **Selected clinical trials of male circumcision**.

**Male circumcision**	**Status**	**Phase**	**Target group**	**Alternate study ID**	**Observation**	**References**
NCT00124878	Unknown	III	Men	22006	No reduction of HIV transmission to female partners	Wamai et al., 2011
NCT00425984	Complete	III	Men	U1AI171-1-02	55% efficacy	Gray et al., 2007
NCT00122525	Terminated	III	Men	ANRS 1265	60% efficacy	Auvert et al., 2005

More information about the individual trials can be found using the NCT number and the following databases: www.aidsinfo.nih.gov/clinical-trials; www.clinicaltrials.gov; http://www.avac.org.

Other alternatives for prevention or cure are targeting the CCR5 receptor as well as the latent HIV reservoir. With regard to the former, the idea of modulating the expression of CCR5 on CD4 T-cells is not new and CCR5 blocking molecules are used in the clinic (e.g., Maraviroc) or have been investigated in animal models or clinical trials (e.g., TAK-779, Aplaviroc, and Vicriviroc). However, resistance, lack of efficiency or toxicity has been associated with these compounds (Ryan, 2005; Sax, 2006; Nichols et al., 2008; Ogert et al., 2008; Demarest et al., 2009; Kitrinos et al., 2009; Wilkin et al., 2010). For example, treatment with CCR5 co-receptor antagonists can lead to selection of resistant viruses whose entry rates into target cells in the presence of the antagonist have been restored to wild-type levels (Putcharoen et al., 2011). Nevertheless, interest in targeting the CCR5 co-receptor persists, in part due to the case study of an individual potentially cured of HIV infection after receiving a bone marrow transplant for treatment of acute myeloid leukemia from a donor homozygous for CCR5Δ32 (Allers et al., 2011). New approaches include attempts to permanently alter the expression of the CCR5 receptor on CD4 cells by gene therapy (e.g., knockout and knockdown) (Cannon and June, 2011). shRNA knockdown of CCR5 in the humanized mouse model has led to reduced expression on T-cells in the gut mucosa, and splenocytes were harder to infect with HIV (Shimizu et al., 2010). In another experiment, RNA interference-mediated knockdown of CCR5 was stable for 10 days and challenged animals exhibited reduced plasma viral loads and enhanced resistance to infection (Kim et al., 2010). Nevertheless, all interventions have drawbacks. Disadvantages of CCR5 knockout include lower anti-tumor, anti-bacterial and anti-parasitic responses, as shown in animal model studies (Gonzalez-Martin et al., 2011; Olive et al., 2011; Sullivan et al., 2011). In addition, disruptions of the normal control of other infectious diseases may occur. For example, in humans CCR5 deficiency is a risk factor for both early and late clinical manifestations of West Nile virus (Lim et al., 2010) and tickborne encephalitis (Kindberg et al., 2008) infections.

The idea of purging the viral reservoir in order to affect a cure of HIV infection has been a long-term goal (Frater, 2011; Palmer et al., 2011). However, simply intensifying drug regimens has not succeeded despite suppression of viral loads below 1 copy/ml plasma (Byakwaga et al., 2011; Dahl et al., 2011). A potential explanation could be on going cell to cell spread of the virus (Sigal et al., 2011). In rare instances upon infection of resting CD4 T-cells HIV goes into latency, a phenomenon known for over 20 years (Bednarik and Folks, 1992). These latent reservoir cells have a long half-life and reactivation leads to reemerging viremia (Iglesias-Ussel and Romerio, 2011; Pace et al., 2011). Mechanisms controlling latency act at the transcriptional level, where hypo-acetylation of histones correlates with repression (Lafeuillade and Stevenson, 2011). Histone deacetylase (HDAC) inhibitors, such as Valproic acid (VPA), a known medication for psychiatric disorders, have been tried together with ART to reduce the latent virus reservoir with mixed results and side-effects at higher doses (Lehrman et al., 2005; Margolis, 2011; Matalon et al., 2011; Routy et al., 2012). The development of safer, more efficient HDAC inhibitors in combination with ART might provide a promising path toward a cure for HIV (Margolis, 2011). Other more specific HDAC inhibitors are under development (Matalon et al., 2011). A promising candidate is Givinostat, shown to be safe in humans, and slated to move into clinical phase II trials (Furlan et al., 2011).

## Vaccine development

HIV vaccines have been designed for both therapy and prevention. Multiple clinical trials with therapeutic vaccines have been conducted. To mention a few, approaches have included rAd5-HIV *gag* (Li et al., 2011) Tat protein or Tat DNA (Caputo et al., 2009), ALVAC encoding multiple HIV genes plus gp160 (Jin et al., 2002), gp160 protein alone (Kundu-Raychaudhuri et al., 2001; Gudmundsdotter et al., 2008), and dendritic cells (DC) pulsed with autologous inactivated virus (García et al., 2011). The overall outcome in most studies was a temporary significant drop in viral loads, induction of cytotoxic T-cell responses, as well as improved CD4 counts. To date, therapeutic vaccines have not progressed to the point of replacing drug therapy. However, this is an important although challenging goal in view of toxicities associated with drug regimens. A key factor in application of all therapeutic vaccines may be early initiation after infection in order to surmount HIV-associated T- and B-cell dysfunctions.

Most of the research emphasis in the field is devoted toward the development of a preventive HIV vaccine. A highly efficacious vaccine would circumvent the need for multiple preventive strategies, and would have its greatest impact in countries of the emerging and developing world where treatment availability is still scarce. However, such a vaccine is still elusive (Girard et al., 2011). The clinical trial (RV144) of ALVAC-HIV recombinant priming followed by gp120 protein boosting (Table 6) (Rerks-Ngarm et al., 2009; Girard et al., 2011) conducted in Thailand showed 31% efficacy. It has been suggested that in Thailand the RV-144 approach could provide modest long term benefit even without revaccination to combat the short-term effectiveness of the vaccine (Schneider et al., 2011). However, the vaccine approach overall was far from reaching the protective efficacy desired. As outlined below, achieving a more efficacious vaccine will require continuing, major research efforts.

**Table 6:** **Selected clinical trials of HIV prophylactic vaccines**.

**Clinical trial identifier**	**Status**	**Phase**	**Vaccine approach**	**Alternative study ID**	**Vaccine components**	**Comment/references**
NCT00002441	Complete	III	Subunit priming and boosting	AIDSVAX B/B, VAX 004	MN rgp120/HIV-1 and GNE8 rgp120/HIV-1	No protection; Flynn et al., 2005
NCT00006327	Complete	III	Subunit priming and boosting	AIDSVAX B/E, VAX 003	MN rgp120/HIV-1 and A244 rgp120/HIV-1	No protection; Pitisuttithum et al., 2006
NCT00223080	Active	III	Vector priming, subunit boosting	RV-144	ALVAC-HIV vCP1521 + AIDSVAX	31.2% efficacy; Rerks-Ngarm et al., 2009
NCT00095576	Terminated	II	Vector priming and boosting	STEP study	Trivalent MRKAd5 HIV-1 gag/pol/nef	No protection; Buchbinder et al., 2008
NCT00413725	Suspended	II	Vector priming and boosting	Phambili study	MRKAd5 HIV-1 gag/pol/nef	No protection; Gray et al., 2011
NCT00865566	Recruiting	II	DNA priming, vector boosting	HVTN 505	VRC-HIVDNA016-00-VP (DNA) + VRC-HIVADV014-00-VP (rAd5)	–
NCT00820846	Active	II	DNA priming, vector boosting	HVTN 205	pGA2/JS7 DNA + MVA/HIV clade B gag-pol-env	–
NCT01418235	Recruiting	I	DNA/vector/subunit	HVTN 086/SAAVI 103	DNA-C2 + MVA-C + gp140 in MF59	–
NCT00062530	Not yet recruiting	I	Recombinant bacteria	P01AI47490	Oral recombinant Salmonella typhi HIV-1 gp120	–
NCT01441193	Recruiting	I	Subunit priming and boosting	ISS P-002	HIV-1 Tat; delta-V2 Env	–
NCT01095224	Recruiting	I	Vector priming and boosting	HVTN 083	rAd35 Env A+rAd5 Env A or rAd5 Env B	–

More information about the individual trials can be found using the NCT number and the following databases: www.aidsinfo.nih.gov/clinical-trials; www.clinicaltrials.gov; http://www.avac.org.

An ideal vaccine should induce long lived memory T-cells with high cytotoxic potential and B-cells able to secrete potent, functional antibodies (Benmira et al., 2010). As T-cell vaccines have been intensively reviewed recently (Ahlers and Belyakov, 2010a,b; Perrin et al., 2010), here we will address B cell immunity. That antibodies alone are sufficient to prevent infection has been clearly shown in passive transfer studies in animal models (Baba et al., 2000; Mascola et al., 2000; Nishimura et al., 2003). Additionally, a recent gene therapy approach using an AAV vector expressing neutralizing antibody conferred protection in non-human primates (Johnson et al., 2009). In addition to the well-known broadly neutralizing antibodies b12, 2G12, 2F5, 4E10, and Z13 (Muster et al., 1993; Burton et al., 1994; Trkola et al., 1996; Zwick et al., 2001) the recent isolation of even more potent broadly neutralizing antibodies from infected patients, including VRC-01, -02, and -03; PG9, PG16, 3BNC117, NIH45-46, PGT121-123, and PGT125-128 (Walker et al., 2009, 2011; Wu et al., 2010; Scheid et al., 2011), shows that the immune system is capable of producing these highly sought antibodies. However, no vaccine approach has been able to elicit them, due in part to the complex structure of the HIV (and SIV) envelope (Roux and Taylor, 2007; Schief et al., 2009; Harris et al., 2011; White et al., 2011). Additionally, the HIV envelope has shown poor binding to germline predecessors of broadly neutralizing antibodies, suggesting a mechanism by which HIV may evade immune responses (Xiao et al., 2009; Chen et al., 2012). In fact, although examination of VRC-01-like antibodies revealed a common pathway of maturation, extensive affinity maturation steps were required to attain broad reactivity (Zhou et al., 2010; Scheid et al., 2011; Wu et al., 2011). Overall, up to 30% of HIV infected individuals develop cross-reactive neutralizing antibodies approximately 3 years after seroconversion, suggesting that the antibodies need multiple rounds of somatic hypermutation and affinity maturation to achieve broad reactivity and that current recombinant envelope immunogens are unable to promote this process from precursor naïve B-cells (Euler et al., 2012).

Reverse vaccinology and structure-based vaccine design approaches focused on known neutralizing antibody targeted envelope epitopes are attempting to develop vaccine immunogens able to elicit the desired neutralizing antibody potency and breadth (Pejchal and Wilson, 2010; Walker and Burton, 2010). One approach to achieve presentation of critical conformational epitopes is stabilization of the CD4-mediated conformational change in the envelope using a single-chain analogue of the HIV gp120-CD4 complex (DeVico et al., 2007). Other approaches as reviewed in Kwong et al. (2011) and Walker and Burton (2010) include virus like particles, soluble trimers, stabilized envelope trimers with deletion of the gp41 transmembrane domain, conformational stabilization of the Env core protein, generation of envelope subdomains (e.g., the outer domain containing the CD4 attachment site), and employment of scaffolds based on informatics and epitope transplantation (e.g., adding the conserved β15 loop of the CD4 binding site to an unrelated structure to present the epitope in its natural configuration). Stable trimers are now available and maintain native conformation (Harris et al., 2011; Lewis et al., 2011; Sellhorn et al., 2011). However, overall envelope glycosylation and the resulting glycan shield can still influence immunogenicity (Benjouad et al., 1992; Kumar et al., 2011) and remains a problem for vaccine development and design (Kwong et al., 2011).

The variable loop regions (V1/V2 and V3) of the viral envelope have regained attention, due to their interaction with the gut-homing α4β7 receptor (Arthos et al., 2008), role in viral transmission, binding to highly potent neutralizing antibodies (Doores and Burton, 2010; Collins-Fairclough et al., 2011; Pejchal et al., 2011) and recent correlation with reduced risk of HIV infection in the RV144 trial (Zolla-Pazner et al., 2011a). As a result, scaffolds that present V1-V2 in the appropriate native conformation are currently under study. However, during HIV infection, V1-V2 loop sequences expand and add glycosylation sites, which in turn could affect binding and neutralization by antibodies (Sagar et al., 2006). The V3 loop, long-thought to elicit only type-specific antibodies, has some structurally conserved elements (Carrow et al., 1991; Boudet et al., 1992; Almond et al., 2010; Jiang et al., 2010), suggesting a potential for inducing broadly neutralizing antibodies. Recently, a V3 scaffold immunogen based on structure-guided design elicited cross-clade neutralizing antibodies in rabbits (Totrov et al., 2010; Zolla-Pazner et al., 2011b), raising hope that similar constructs would be equally effective in humans. Nevertheless, structural approaches and reverse engineering still face multiple obstacles and limitations from numerous extrinsic factors such as the host immunoglobulin gene repertoire and cellular regulatory mechanisms in the immunized host during antigen processing and antibody affinity maturation (Van Regenmortel, 2011, 2012). Additional limitations are imposed by discontinuous epitopes that are difficult to isolate in their native tertiary structure, and quaternary structure which introduces transient, conformational “neotopes” and masked “cryptotopes” which appear only in the context of the assembled virus particle (Van Regenmortel, 2011, 2012). Cross-reactivity or heterospecificity arising from weak antigen B-cell receptor interactions can also potentially lead to auto reactive antibodies as reported for 2F5 and 4E10 (Haynes et al., 2005). Understanding the complex interactions between antigen presenting cells (APC) and B-cells at different mucosal sites or in lymph nodes is important for designing optimal vaccine approaches. The HIV envelope in particular is poorly immunogenic. It can suppress CD4 T-cell activation and proliferation (Fernando et al., 2007) and can mediate T-cell apoptosis (Arthos et al., 2002). Further, high affinity antibodies recognizing the CD4-binding site can skew MHC class II presentation by partially obstructing antigen processing, leading to decreased anti-gp120 T helper responses (Tuen et al., 2005). It is crucial to find measures to boost the envelope's immunogenicity in order to elicit potent and durable anti-env responses. The contributions of cells of the innate immune system, such as NK, NKT, γδ T-cells, and DC are under extensive investigation (Bostik et al., 2010; Palucka et al., 2010; Agrati et al., 2011; Altfeld et al., 2011; Padte et al., 2011; Ansari et al., 2012). Cross-talk between the innate and adaptive immune systems is important in shaping adaptive B-cell responses (Cerutti et al., 2011). Innate programming of protective immunity in general and programming of antibody (and T cell) responses by innate immune components have recently been reviewed (Pulendran and Ahmed, 2011).

Apart from design of specific vaccine components and regimens, the route of administration can influence immune response elicitation, systemically and mucosally at genital/rectal sites (Demberg and Robert-Guroff, 2009; Mestecky et al., 2010). Vectored vaccines can be administered by a variety of routes and in addition to eliciting T cell responses also effectively induce humoral immunity. Prominent vectors currently in use are replication competent or defective Adenoviruses, Canarypox (ALVAC), and Vaccinia (NYVAC, MVA) (Franchini et al., 2004; Gomez et al., 2008; Patterson and Robert-Guroff, 2008; Barouch, 2010; Chen et al., 2010; Rollier et al., 2011; Weli and Tryland, 2011). Adenoviruses, by targeting mucosal epithelium, induce mucosal as well as systemic immunity. In fact, replication-competent Adenovirus vectors display a broad biodistribution. Regardless of mucosal immunization route, they target and persist in macrophages and myeloid dendritic cells (Patterson et al., 2012) and elicit comparable immune responses systemically and mucosally (Xiao et al., 2012). An interesting approach is the use of recombinant Cytomegalovirus (CMV) vectors. These recombinants also persist in the host and stimulate strong CD4 and CD8 cell responses (Hansen et al., 2011). Using rhesus CMV vectors expressing SIV transgenes, strong protection from acquisition of SIV_mac239_ was achieved in a rectal low-dose challenge model (Hansen et al., 2011). In spite of the impressive protection in rhesus macaques, translation into the clinic may present a challenge due to the significant morbidity that can be caused by reoccurring CMV infection, seen for example, in immunosuppressed individuals (Sellar and Peggs, 2012). Other vectors targeting the mucosa include integrase-defective lentiviruses (Negri et al., 2011), noroviruses (Herbst-Kralovetz et al., 2010) and engineered bacteria such as Listeria, Salmonella, Lactococcus, and Lactobacillus (Shahabi et al., 2010; Bahey-El-Din and Gahan, 2011; Lagenaur et al., 2011).

DNA vaccines alone can also successfully prime and boost the immune system (Ferraro et al., 2011; Sardesai and Weiner, 2011) and together with viral vectors have induced lasting immune responses and long-term control of virus infection following challenge of non-human primates (Patel et al., 2010; Lai et al., 2011; Manrique et al., 2011; Winstone et al., 2011). Electroporation, instead of intramuscular injection of DNA, has enhanced development of durable immunity (Patel et al., 2010). Moreover, use of DNA vaccines facilitates incorporation of cytokine immune modulators (IL-15, IL-12, GM-CSF, and TNF-α) into the vaccine strategy (Demberg et al., 2008; Ferraro et al., 2011; Lai et al., 2011; Manrique et al., 2011; Winstone et al., 2011).

Vectored vaccines are often combined with envelope protein boosts, requiring an adjuvant. Cytokines and TLR ligands can serve this purpose (Berzofsky et al., 2001; Tovey and Lallemand, 2010; Bode et al., 2011; Duthie et al., 2011) but traditionally, adjuvants function as depots while also acting as immunostimulants (Reed et al., 2009; Carter and Reed, 2010). Currently there are only two adjuvants in licensed vaccines available in the USA: aluminum salts, including aluminum hydroxide, aluminum phosphate and alum (potassium aluminum sulfate); and AS04 (aluminum hydroxide combined with monophosphoryl lipid (http://www.fda.gov/BiologicsBloodVaccines/Safety Availability/VaccineSafety/ucm187810.htm). In Europe, additional approved adjuvants include MF59 (squalene-based oil in water emulsion) and AS03 (also squalene-based) (Tritto et al., 2009; Carter and Reed, 2010; O'Hagan et al., 2011). Yet additional adjuvants are needed. Broad new candidates include carbohydrate-based and saponin based compounds, ISCOM Matrix, cationic liposomes, and Nano-microparticles (Oyewumi et al., 2010; Christensen et al., 2011; Lovgren Bengtsson et al., 2011; Petrovsky and Cooper, 2011; Ragupathi et al., 2011; Schijns and Lavelle, 2011).

A crucial aspect for the evaluation of candidate vaccines is availability of relevant animal models. Currently there are only two appropriate models: non-human primates and the newly developed bone marrow/liver/thymus (BLT) mouse model. The BLT mouse develops human T cells and B cells, monocyte/macrophages and DC (Wege et al., 2008). In this model HIV latency has been described and thus the BLT mouse is suitable for testing drug regimens, including evaluation of HDAC inhibitors (Marsden et al., 2011). Due to the reconstitution of the female BLT mouse reproductive tract with human immune cells, vaginal challenges can be performed and microbicides can be evaluated (Denton et al., 2011; Olesen et al., 2011). In addition, candidate vaccines based on non-replicating vectors as well as novel adjuvants can be tested in a shorter time frame and at lower cost compared to non-human primate models (Biswas et al., 2011). However, the BLT mouse model has its limitations, including lack of proper T-cell help which affects development of humoral responses (Biswas et al., 2011).

Overall non-human primates provide the best, although not perfect, model for evaluation of pre-clinical candidate vaccines (Morgan et al., 2008; Rompay, 2011). The SIV rhesus macaque system appropriately models HIV disease of humans with regard to mode of transmission, viremia levels, depletion of CD4 cells, and development of AIDS. However, the difference in the SIV and HIV envelopes does not allow evaluation of anti-HIV envelope immunity in the SIV system. The SHIV model while not as robust, allows evaluation of vaccines targeting the HIV envelope and their ability to elicit sterilizing immunity, however, it is generally poorly pathogenic and overall a poor model of HIV biology and disease progression in humans. Further, experiments in non-human primates are expensive, and the number of macaques required for a pre-clinical vaccine study has increased with the use of low-dose repeated challenge experiments that better mimic the HIV infection of people (Keele et al., 2009). Thus, evaluation of a single vaccine candidate and a control could require 50 macaques (Hudgens and Gilbert, 2009), However, it could be designed and completed quickly. The cost for such a macaque study is far surpassed by that of a human clinical trial. As summarized by Shedlock et al. (2009), the non-human primate model has a key role to play in evaluating vaccine candidates for the ability to induce improved immune responses, in testing hypothesis driven research, in providing proof of concept, and in identifying new immune targets for incorporation into vaccine design. In that way the model helps select vaccine candidates for advancement into the limited number of possible clinical trials.

## Summary and conclusion

As briefly summarized by this review the substantial progress made in AIDS treatment has stimulated the concept of “treatment as prevention.” Additionally, vaccine development has picked up momentum due to new discoveries and the modest success of the RV144 clinical trial in Thailand. However, to achieve global control of HIV/AIDS infection in the absence of a highly efficacious vaccine or cure, a combination of multiple preventive measures will need to be continuously applied. Further investigation of all elements of the host immune system, both innate and adaptive, as well as development of novel treatment strategies should proceed in order to insure strong control of the AIDS pandemic and eventual eradication of the disease.

### Conflict of interest statement

The authors declare that the research was conducted in the absence of any commercial or financial relationships that could be construed as a potential conflict of interest.
